# Crystal structure of [5-bromo-2-(pyridin-2-yl-*κN*)phenyl-*κC* ^1^](pentane-2,4-dionato-*κ* ^2^ *O*,*O*′)platinum(II)

**DOI:** 10.1107/S2056989015017478

**Published:** 2015-09-30

**Authors:** Keito Fukuda, Tomoaki Sugaya, Koji Ishihara

**Affiliations:** a Department of Chemistry and Biochemistry, School of Advanced Science and Engineering, Waseda University, Okubo, Shinjuku-ku, Tokyo 169-8555, Japan

**Keywords:** crystal structure, platinum(II), cyclo­metalated complex, acetyl­acetonato ligand, π–π inter­actions

## Abstract

The title cyclo­metalated platinum(II) complex with 2-(4-bromo­phen­yl)pyridinato and acetyl­acetonato ligands, [Pt(C_11_H_7_BrN)(C_5_H_7_O_2_)], consists of two crystallographically non-equivalent dimers, each stacked by π–π inter­actions with distances of ≃ 3.4 Å. In both dimers, the platinum(II) complexes are arranged anti­parallel to each other. Each complex exhibits a slightly distorted square-planar coordination environment around the central Pt(II) atom. The dihedral angles between two chelate rings including the Pt^II^ atom in these complexes are 0.08 (12) and 1.54 (9)°.

## Abstract 2

In the title complex, [Pt(C_11_H_7_BrN)(C_5_H_7_O_2_)], two crystallographically non-equivalent dimers stacked by π–π inter­actions are arranged anti­parallel to each other.

## Chemical context   

Square-planar cyclo­metalated platinum(II) complexes with luminescent properties have recently attracted attention because of their potential applications (Chi & Chou, 2010 ▸; Ma *et al.*, 2013 ▸), such as DNA probing, as chemical sensors or as organic light-emitting diodes (OLEDs). In particular, platinum(II) complexes including *β*-diketonate anions (*e.g.* acetyl­acetonate) as an ancillary ligand have been widely studied because of their excellent stabilities and high quantum yields. Although these complexes afford luminescence in the solid state, their crystal structures have not been sufficiently explored. We report herein the crystal structure of the cyclo­metalated platinum(II) complex with 2-(4-bromo­phen­yl)pyridinato (Brppy, C_11_H_7_BrN) and acetyl­acetonato (acac, C_5_H_7_O_2_) ligands, [Pt(Brppy)(acac)].
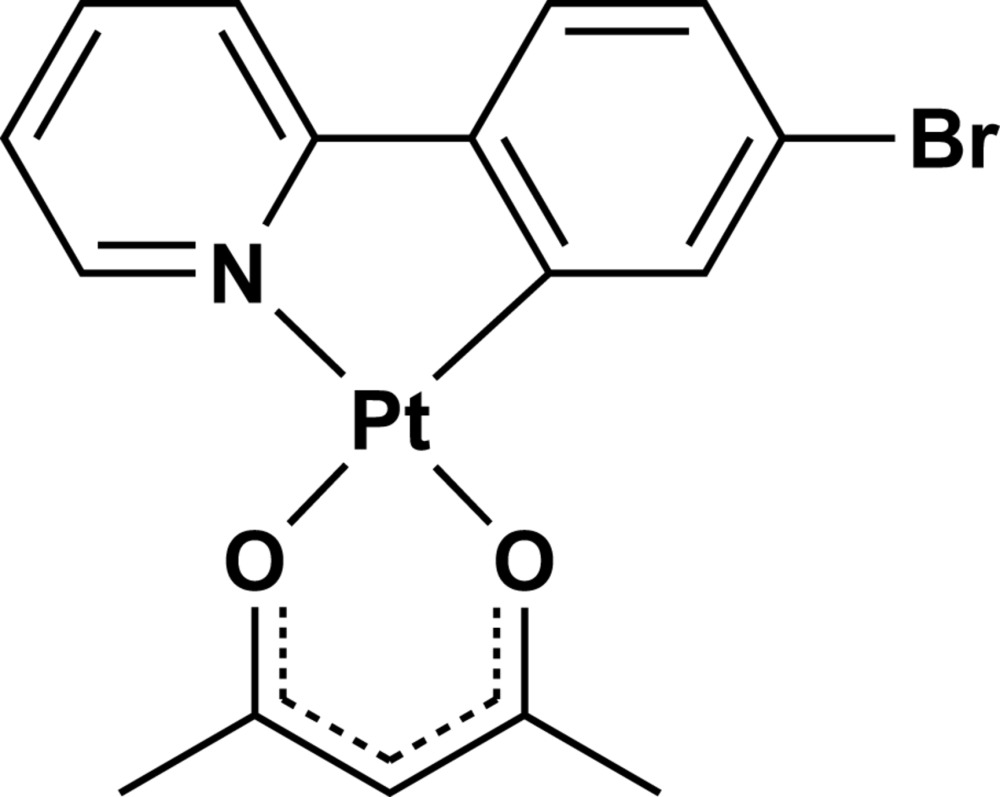


## Structural commentary   

**Figure 1:**
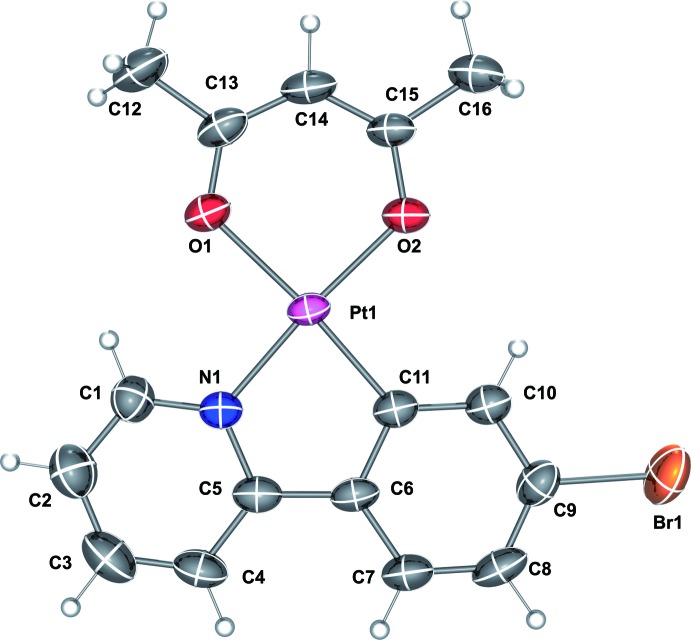
Mol­ecular structure of one of the two independent Pt^II^ complexes of the title compound, with displacement ellipsoids drawn at the 50% probability level.

The asymmetric unit of the title compound contains two complex mol­ecules with very similar configurations (r.m.s. deviation of fit of two molecules = 0.07 Å). The structure of one of the complex mol­ecules of the title compound is shown in Fig. 1 ▸. In both complexes, the Pt^II^ atom is coordinated by C and N atoms of the bidentate Brppy ligand and two O atoms of the acac ligand. The coordination environments around the central Pt^II^ atoms (Pt1 and Pt2) are slightly distorted from an ideal square-planar configuration, with angles around Pt1 in the range 81.89 (18)–93.04 (17)° and around Pt2 in the range 81.73 (18)–93.57 (16)°. The Pt—C bond lengths [Pt1—C11 = 1.970 (5) and Pt2—C27 = 1.969 (5) Å] are slightly shorter than the Pt—N bond lengths [Pt1—N1 = 1.995 (4) and Pt2—N2 = 1.999 (4) Å] due to the stronger electron-donating ability of a C atom compared to that of an N atom. Pt—O bond lengths are compiled in Table 1 ▸. The phenyl and pyridyl rings are approximately coplanar [the dihedral angle between the N1,C1–C5 and C6–C11 rings is 1.31 (17)° while that between the N2,C17–C21 and C22–C27 rings is 3.12 (13)°]. In addition, the dihedral angles between two planes composed of the two chelate rings in the cyclo­metalated complex are 0.08 (12)° (involving Pt1) and 1.54 (9)° (involving Pt2).

## Supra­molecular features   

**Figure 2:**
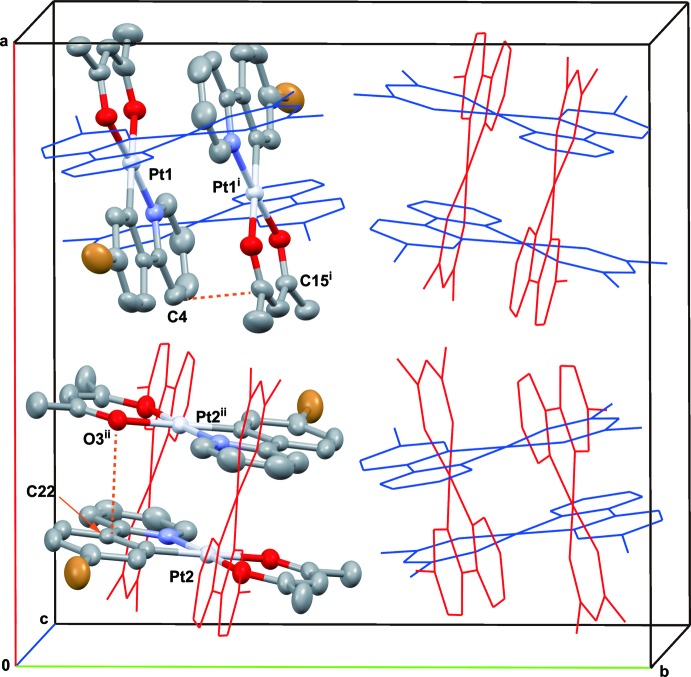
Crystal packing of the title complex, viewed perpendicular to the *ab* plane. Dashed lines represent the shortest inter­molecular contacts. Red wires represent the Pt1 mol­ecule, and blue wires the Pt2 mol­ecule. H atoms are omitted for clarity. [Symmetry codes: (i) –*x* + 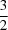, –*y* + 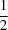, –*z* + 1; (ii) –*x* + 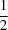, –*y* + 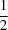, –*z* + 1.]

As shown in Figs. 2 ▸ and 3 ▸, in the unit cell two non-equivalent dimers are formed by π–π inter­actions between individual complexes. Each non-equivalent dimer is in a head-to-tail form. In each unit cell both types of head-to-tail dimers stacked with an inter­molecular π–π inter­action are perpendicular to each other. The π-plane of one Pt^II^ complex (Pt1) is directed to the *b* axis, on the other hand, that of the other complex (Pt2) is directed to the *a* axis. The shortest inter­molecular contacts are C4⋯C15^i^ = 3.406 (7) and C22⋯O3^ii^ = 3.402 (6) Å [symmetry codes: (i) –*x* + 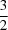, –*y* + 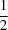, −*z* + 1; (ii) –*x* + 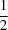, –*y* + 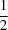, –*z* + 1]. Weak C—H⋯O and C—H⋯Br inter­actions might also help to consolidate the crystal packing (Table 2 ▸). There is almost no inter­action between the two Pt^II^ atoms in each dimers because the *z*-axes of Pt1 and Pt2 are not coaxial. In fact, the Pt—Pt contacts [Pt1⋯Pt1^i^ = 3.688 (1) and Pt2⋯Pt2^ii^ = 3.723 (1) Å] are longer than the van der Waals diameter of the Pt atom (3.5 Å; Bondi, 1964 ▸)

## Synthesis and crystallization   

The title complex was synthesized according to a traditional two-step preparation method *via* the di­chlorido-bridged dimer complex [Pt(C_11_H_7_BrN)(*μ*-Cl)]_2_ (Cockburn *et al.*, 1973 ▸; Liu *et al.*, 2009 ▸), though one-pot synthesis has been reported recently (Hudson *et al.*, 2012 ▸).

**[Pt(C_11_H_7_BrN)(** ***μ*** **-Cl)]_2_:** A mixture of 2-(4-bromo­phen­yl)pyridine (0.585 g, 2.5 mmol) and K_2_PtCl_4_ (1.00 g, 2.4 mmol) in a 2-eth­oxy­ethanol–water mixture (45 ml/15 ml) was stirred for 6 h at 333 K under an Ar atmosphere. After cooling to room temperature, the yellow–green precipitate was filtered off, washed with di­chloro­methane, and dried *in vacuo*. Yield: 0.535 g, (48.2%).

**[Pt(C_11_H_7_BrN)(C_5_H_7_O_2_)]:** A mixture of the di­chlorido-bridged dimer complex (0.185 g, 0.20 mmol), acetyl­acetone (0.020 g, 0.20 mmol) and Na_2_CO_3_ (0.211 g, 2.0 mmol) in 2-eth­oxy­ethanol was stirred for 7 h at 323 K under an Ar atmosphere. After cooling to room temperature, the yellow precipitate was filtered off and dried *in vacuo*. Yield: 0.200 g (47.6%)

Yellow single crystals suitable for X-ray structural analysis were grown by vapor diffusion of hexane into the di­chloro­methane solution of the title complex.

Analysis found (calculated for C_16_H_14_BrNO_2_Pt): C, 36.15 (36.45); H, 2.25 (2.68); N, 2.59 (2.66). UV–vis [CHCl_3_, *λ*_max_ nm^−1^ (*∊* / L mol^−1^ cm^−1^)]: 262 (29800), 280 (27500), 317 (*sh*, 11700), 330 (*sh*, 9400), 363 (6400), 389 (4200). ^1^H NMR (CDCl_3_, 298 K); 8.97 (*d*, *J*_Pt-H_ = 40.0 Hz, *J* = 6.0 Hz, 1H), 7.81 (*t*, *J* = 6.0 Hz, 1H), 7.71 (*s*, *J*_Pt-H_ = 40.0 Hz, 1H), 7.57 (*d*, *J* = 6.0 Hz, 1H), 7.31-7.45 (*m*, 2H), 7.14 (*t*, *J* = 6.0 Hz, 1H), 5.48 (*s*, 1H), 2.03 (s, 3H), 2.01 (*s*, 3H).

## Refinement   

Crystal data, data collection and structure refinement details are summarized in Table 3 ▸. All H atoms were placed in geometrically idealized positions and refined using a riding model, with C—H = 0.95 Å, *U*_iso_(H) = 1.2*U*_eq_(C) for C*sp* ^2^–H, and *U*_iso_(H) = 1.5*U*_eq_(C) for methyl H atoms.

## Supplementary Material

Crystal structure: contains datablock(s) I. DOI: 10.1107/S2056989015017478/wm5214sup1.cif

Structure factors: contains datablock(s) I. DOI: 10.1107/S2056989015017478/wm5214Isup2.hkl

Supporting information file. DOI: 10.1107/S2056989015017478/wm5214Isup3.tif

CCDC reference: 1425736

Additional supporting information:  crystallographic information; 3D view; checkCIF report

## Figures and Tables

**Figure 3:**
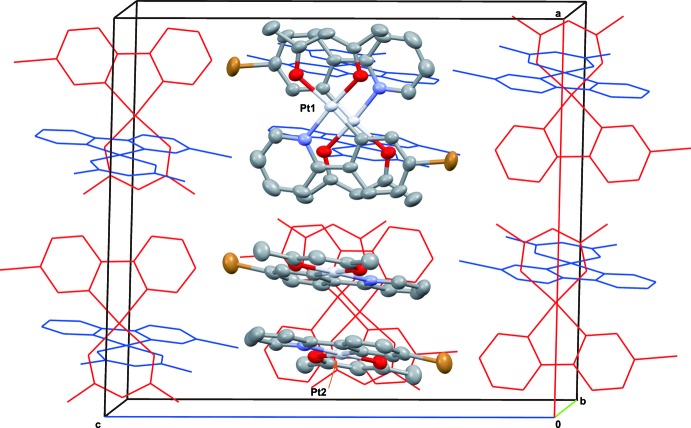
Crystal packing of the title complex, viewed perpendicular to the *ac* plane. Red wires represent the Pt1 mol­ecule, and blue wires the Pt2 mol­ecule. H atoms are omitted for clarity.

**Table 1:** Selected bond lengths ()

O1Pt1	2.077(3)	O3Pt2	2.081(3)
O2Pt1	2.007(3)	O4Pt2	2.005(3)

**Table 2:** Hydrogen-bond geometry (, )

*D*H*A*	*D*H	H*A*	*D* *A*	*D*H*A*
C1H1O1	0.95	2.40	2.999(7)	121
C4H4O4^i^	0.95	2.58	3.281(6)	131
C17H17O3	0.95	2.45	3.034(6)	120
C17H17Br1^ii^	0.95	2.87	3.693(6)	145

Symmetry codes: (i) 
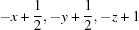
; (ii) 
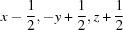
.

**Table 3:** Experimental details

Crystal data	
Chemical formula	[Pt(C_11_H_7_BrN)(C_5_H_7_O_2_)]
*M*_r_	527.28
Crystal system, space group	Monoclinic, *C*2/*c*
Temperature (K)	200
*a*, *b*, *c* ()	17.557(2), 17.876(2), 19.832(2)
()	91.397(1)
*V* (^3^)	6222.4(13)
*Z*	16
Radiation type	Mo *K*
(mm^1^)	11.59
Crystal size (mm)	0.18 0.06 0.02

Data collection	
Diffractometer	Bruker APEXII CCD area detector
Absorption correction	Multi-scan (*SADABS*; Bruker, 2014 ▸)
*T*_min_, *T*_max_	0.48, 0.80
No. of measured, independent and observed [*I* > 2(*I*)] reflections	35025, 7103, 6001
*R*_int_	0.038
(sin /)_max_ (^1^)	0.649

Refinement	
*R*[*F* ^2^ > 2(*F* ^2^)], *wR*(*F* ^2^), *S*	0.027, 0.070, 1.01
No. of reflections	7103
No. of parameters	383
H-atom treatment	H-atom parameters constrained
_max_, _min_ (e ^3^)	3.66, 1.20

Computer programs: *APEX2* and *SAINT* (Bruker, 2014 ▸), *SHELXS97* (Sheldrick, 2008 ▸), *SHELXL2014* (Sheldrick, 2015 ▸), *ORTEP-3 for Windows* (Farrugia, 2012 ▸) and *Mercury* (Macrae *et al.*, 2008 ▸).

## supplementary crystallographic information

### Crystal data

**Table:**

[Pt(C_11_H_7_BrN)(C_5_H_7_O_2_)]	*F*(000) = 3936
*M**_r_* = 527.28	*D*_x_ = 2.251 Mg m^−^^3^
Monoclinic, *C*2/*c*	Mo *K*α radiation, λ = 0.71073 Å
*a* = 17.557 (2) Å	Cell parameters from 9927 reflections
*b* = 17.876 (2) Å	θ = 2.3–27.3°
*c* = 19.832 (2) Å	µ = 11.59 mm^−^^1^
β = 91.397 (1)°	*T* = 200 K
*V* = 6222.4 (13) Å^3^	Lath, yellow
*Z* = 16	0.18 × 0.06 × 0.02 mm

### Data collection

**Table:**

Bruker APEXII CCD area detector diffractometer	7103 independent reflections
Radiation source: Bruker TXS fine-focus rotating anode	6001 reflections with *I* > 2σ(*I*)
Bruker Helios multilayer confocal mirror monochromator	*R*_int_ = 0.038
Detector resolution: 8.333 pixels mm^-1^	θ_max_ = 27.5°, θ_min_ = 1.6°
phi and ω scans	*h* = −22→22
Absorption correction: multi-scan (*SADABS*; Bruker, 2014)	*k* = −23→23
*T*_min_ = 0.48, *T*_max_ = 0.80	*l* = −25→25
35025 measured reflections	

### Refinement

**Table:**

Refinement on *F*^2^	0 restraints
Least-squares matrix: full	Hydrogen site location: inferred from neighbouring sites
*R*[*F*^2^ > 2σ(*F*^2^)] = 0.027	H-atom parameters constrained
*wR*(*F*^2^) = 0.070	*w* = 1/[σ^2^(*F*_o_^2^) + (0.0368*P*)^2^ + 16.0306*P*] where *P* = (*F*_o_^2^ + 2*F*_c_^2^)/3
*S* = 1.01	(Δ/σ)_max_ = 0.001
7103 reflections	Δρ_max_ = 3.66 e Å^−^^3^
383 parameters	Δρ_min_ = −1.19 e Å^−^^3^

### Special details

**Table:**

Geometry. Distance SDEV3.4016 (0.0055) C22 - O3_$6 3.4056 (0.0070) C4 - C15_$5 3.6879 (0.0005) Pt1 - Pt1_$5 3.7230 (0.0005) Pt2 - Pt2_$6$5 1.5 - *x*, 0.5 - *y*, 1 - *z* $6 0.5 - *x*, 0.5 - *y*, 1 - *z*Least-squares planes (*x*,*y*,*z* in crystal coordinates) and deviations from them (* indicates atom used to define plane)17.0464 (0.0086) *x* + 3.5524 (0.0343) *y* - 3.1157 (0.0389) *z* = 1.9362 (0.0174)* -0.0159 (0.0032) C22 * 0.0106 (0.0035) C23 * 0.0050 (0.0037) C24 * -0.0152 (0.0036) C25 * 0.0096 (0.0034) C26 * 0.0059 (0.0032) C27Rms deviation of fitted atoms = 0.011217.1816 (0.0082) *x* + 3.3837 (0.0389) *y* - 2.0681 (0.0436) *z* = 2.4419 (0.0278)Angle to previous plane (with approximate e.s.d.) = 3.118 (0.129)* -0.0040 (0.0032) N2 * 0.0023 (0.0037) C17 * 0.0000 (0.0041) C18 * -0.0004 (0.0041) C19 * -0.0014 (0.0038) C20 * 0.0036 (0.0033) C21Rms deviation of fitted atoms = 0.00253.5018 (0.0341) *x* + 17.1159 (0.0108) *y* - 4.2313 (0.0388) *z* = 3.1261 (0.0262)Angle to previous plane (with approximate e.s.d.) = 66.846 (0.177)* 0.0047 (0.0032) C6 * -0.0001 (0.0036) C7 * -0.0055 (0.0037) C8 * 0.0064 (0.0036) C9 * -0.0015 (0.0033) C10 * -0.0040 (0.0031) C11Rms deviation of fitted atoms = 0.00433.8621 (0.0359) *x* + 17.0726 (0.0113) *y* - 4.0479 (0.0426) *z* = 3.4669 (0.0355)Angle to previous plane (with approximate e.s.d.) = 1.309 (0.166)* -0.0056 (0.0031) N1 * 0.0048 (0.0038) C1 * -0.0003 (0.0042) C2 * -0.0032 (0.0042) C3 * 0.0024 (0.0037) C4 * 0.0019 (0.0032) C5Rms deviation of fitted atoms = 0.003517.0575 (0.0059) *x* + 3.8901 (0.0226) *y* - 2.3217 (0.0295) *z* = 2.4371 (0.0163)Angle to previous plane (with approximate e.s.d.) = 63.889 (0.149)* 0.0136 (0.0026) O3 * 0.0004 (0.0034) C29 * -0.0121 (0.0038) C30 * -0.0006 (0.0035) C31 * 0.0149 (0.0026) O4 * -0.0162 (0.0018) Pt2Rms deviation of fitted atoms = 0.011717.1494 (0.0057) *x* + 3.4219 (0.0232) *y* - 2.3782 (0.0383) *z* = 2.2709 (0.0209)Angle to previous plane (with approximate e.s.d.) = 1.538 (0.086)* -0.0073 (0.0024) N2 * 0.0020 (0.0029) C21 * 0.0076 (0.0030) C22 * -0.0104 (0.0026) C27 * 0.0081 (0.0018) Pt2Rms deviation of fitted atoms = 0.00763.7521 (0.0218) *x* + 17.0640 (0.0077) *y* - 4.2225 (0.0285) *z* = 3.2745 (0.0235)Angle to previous plane (with approximate e.s.d.) = 65.705 (0.111)* -0.0132 (0.0026) O1 * -0.0080 (0.0035) C13 * 0.0257 (0.0037) C14 * -0.0103 (0.0034) C15 * -0.0121 (0.0026) O2 * 0.0179 (0.0017) Pt1Rms deviation of fitted atoms = 0.01573.7578 (0.0215) *x* + 17.0576 (0.0090) *y* - 4.2485 (0.0367) *z* = 3.2795 (0.0249)Angle to previous plane (with approximate e.s.d.) = 0.080 (0.123)* -0.0089 (0.0023) N1 * 0.0120 (0.0028) C5 * -0.0086 (0.0028) C6 * 0.0027 (0.0024) C11 * 0.0028 (0.0017) Pt1Rms deviation of fitted atoms = 0.0079

### Fractional atomic coordinates and isotropic or equivalent isotropic displacement parameters (Å^2^)

**Table:**

	*x*	*y*	*z*	*U*_iso_*/*U*_eq_	
Br1	0.63794 (4)	0.11449 (4)	0.24876 (3)	0.06869 (19)	
Br2	0.13287 (5)	0.08166 (4)	0.25166 (3)	0.06997 (19)	
C1	0.6968 (3)	0.1984 (3)	0.6440 (3)	0.0478 (12)	
H1	0.7459	0.1928	0.6646	0.057*	
C2	0.6367 (4)	0.2211 (3)	0.6835 (3)	0.0614 (16)	
H2	0.6441	0.2304	0.7304	0.074*	
C3	0.5655 (4)	0.2296 (3)	0.6522 (3)	0.0626 (17)	
H3	0.5231	0.2449	0.6777	0.075*	
C4	0.5565 (3)	0.2159 (3)	0.5846 (3)	0.0484 (13)	
H4	0.5080	0.2221	0.5632	0.058*	
C5	0.6187 (3)	0.1929 (2)	0.5470 (3)	0.0368 (10)	
C6	0.6190 (3)	0.1739 (2)	0.4758 (3)	0.0355 (10)	
C7	0.5543 (3)	0.1762 (3)	0.4328 (3)	0.0467 (12)	
H7	0.5065	0.1904	0.4502	0.056*	
C8	0.5596 (3)	0.1583 (3)	0.3660 (3)	0.0493 (14)	
H8	0.5160	0.1593	0.3367	0.059*	
C9	0.6299 (3)	0.1388 (3)	0.3424 (3)	0.0455 (12)	
C10	0.6949 (3)	0.1351 (3)	0.3833 (2)	0.0392 (11)	
H10	0.7421	0.1207	0.3649	0.047*	
C11	0.6905 (3)	0.1526 (2)	0.4509 (2)	0.0342 (10)	
C12	0.9693 (4)	0.1381 (4)	0.6572 (3)	0.0652 (17)	
H12A	0.9552	0.1822	0.6835	0.098*	
H12B	1.0234	0.1410	0.6463	0.098*	
H12C	0.9602	0.0928	0.6837	0.098*	
C13	0.9223 (3)	0.1355 (3)	0.5936 (3)	0.0445 (12)	
C14	0.9569 (3)	0.1148 (3)	0.5327 (3)	0.0475 (13)	
H14	1.0104	0.1067	0.5349	0.057*	
C15	0.9213 (3)	0.1051 (3)	0.4705 (3)	0.0395 (11)	
C16	0.9671 (3)	0.0762 (3)	0.4126 (3)	0.0514 (14)	
H16A	0.9435	0.0305	0.3947	0.077*	
H16B	1.0192	0.0653	0.4286	0.077*	
H16C	0.9684	0.1142	0.3770	0.077*	
C17	0.1737 (3)	0.2277 (3)	0.6336 (3)	0.0477 (13)	
H17	0.1651	0.2790	0.6435	0.057*	
C18	0.1893 (3)	0.1793 (4)	0.6855 (3)	0.0591 (15)	
H18	0.1913	0.1967	0.7308	0.071*	
C19	0.2023 (3)	0.1049 (4)	0.6714 (3)	0.0629 (17)	
H19	0.2133	0.0706	0.7069	0.075*	
C20	0.1990 (3)	0.0805 (3)	0.6053 (3)	0.0511 (13)	
H20	0.2077	0.0293	0.5951	0.061*	
C21	0.1831 (3)	0.1312 (3)	0.5538 (3)	0.0411 (11)	
C22	0.1765 (2)	0.1157 (3)	0.4814 (3)	0.0377 (11)	
C23	0.1874 (3)	0.0452 (3)	0.4521 (3)	0.0452 (12)	
H23	0.2023	0.0040	0.4796	0.054*	
C24	0.1767 (3)	0.0357 (3)	0.3844 (3)	0.0482 (13)	
H24	0.1836	−0.0121	0.3645	0.058*	
C25	0.1556 (3)	0.0967 (3)	0.3451 (3)	0.0444 (12)	
C26	0.1472 (3)	0.1677 (3)	0.3722 (3)	0.0410 (11)	
H26	0.1346	0.2089	0.3438	0.049*	
C27	0.1574 (3)	0.1782 (3)	0.4412 (3)	0.0360 (10)	
C28	0.1093 (4)	0.4917 (3)	0.5815 (3)	0.0568 (15)	
H28A	0.0791	0.4731	0.6189	0.085*	
H28B	0.0838	0.5352	0.5611	0.085*	
H28C	0.1600	0.5062	0.5985	0.085*	
C29	0.1167 (3)	0.4308 (3)	0.5292 (3)	0.0435 (12)	
C30	0.1027 (3)	0.4486 (3)	0.4614 (3)	0.0490 (13)	
H30	0.0888	0.4990	0.4520	0.059*	
C31	0.1067 (3)	0.4011 (3)	0.4063 (3)	0.0433 (12)	
C32	0.0874 (4)	0.4303 (3)	0.3369 (3)	0.0617 (16)	
H32A	0.1263	0.4141	0.3055	0.093*	
H32B	0.0855	0.4851	0.3379	0.093*	
H32C	0.0376	0.4108	0.3219	0.093*	
N1	0.6882 (2)	0.1843 (2)	0.5787 (2)	0.0349 (8)	
N2	0.1701 (2)	0.2051 (2)	0.5697 (2)	0.0368 (9)	
O1	0.85200 (19)	0.15218 (18)	0.59970 (18)	0.0418 (8)	
O2	0.85083 (18)	0.11665 (19)	0.45482 (17)	0.0402 (8)	
O3	0.13515 (19)	0.36673 (18)	0.55181 (18)	0.0410 (8)	
O4	0.1237 (2)	0.33092 (18)	0.40711 (17)	0.0400 (8)	
Pt1	0.77288 (2)	0.15171 (2)	0.52011 (2)	0.03211 (6)	
Pt2	0.14668 (2)	0.27243 (2)	0.49142 (2)	0.03308 (6)	

### Atomic displacement parameters (Å^2^)

**Table:**

	*U*^11^	*U*^22^	*U*^33^	*U*^12^	*U*^13^	*U*^23^
Br1	0.0827 (5)	0.0827 (5)	0.0401 (3)	−0.0135 (4)	−0.0115 (3)	0.0119 (3)
Br2	0.0957 (5)	0.0599 (4)	0.0547 (4)	−0.0007 (3)	0.0093 (3)	−0.0143 (3)
C1	0.052 (3)	0.043 (3)	0.047 (3)	0.001 (2)	0.000 (2)	−0.003 (2)
C2	0.071 (4)	0.056 (4)	0.057 (4)	0.003 (3)	0.008 (3)	−0.005 (3)
C3	0.064 (4)	0.052 (4)	0.073 (4)	0.006 (3)	0.027 (3)	−0.001 (3)
C4	0.036 (3)	0.039 (3)	0.070 (4)	0.006 (2)	0.009 (3)	0.006 (3)
C5	0.032 (2)	0.023 (2)	0.056 (3)	−0.0007 (18)	0.001 (2)	0.009 (2)
C6	0.027 (2)	0.026 (2)	0.053 (3)	−0.0041 (18)	−0.004 (2)	0.009 (2)
C7	0.031 (3)	0.049 (3)	0.060 (3)	0.000 (2)	−0.004 (2)	0.008 (3)
C8	0.038 (3)	0.045 (3)	0.063 (4)	−0.007 (2)	−0.019 (3)	0.017 (3)
C9	0.050 (3)	0.046 (3)	0.040 (3)	−0.008 (2)	−0.007 (2)	0.009 (2)
C10	0.037 (3)	0.035 (2)	0.046 (3)	−0.004 (2)	−0.001 (2)	0.009 (2)
C11	0.033 (2)	0.024 (2)	0.045 (3)	−0.0028 (18)	−0.002 (2)	0.0079 (19)
C12	0.048 (4)	0.079 (5)	0.068 (4)	−0.001 (3)	−0.020 (3)	0.008 (3)
C13	0.036 (3)	0.043 (3)	0.054 (3)	−0.008 (2)	−0.011 (2)	0.014 (2)
C14	0.028 (2)	0.046 (3)	0.068 (4)	0.003 (2)	−0.006 (2)	0.013 (3)
C15	0.027 (2)	0.040 (3)	0.051 (3)	0.001 (2)	0.001 (2)	0.013 (2)
C16	0.035 (3)	0.052 (3)	0.067 (4)	0.004 (2)	0.006 (2)	0.014 (3)
C17	0.041 (3)	0.051 (3)	0.051 (3)	−0.001 (2)	0.000 (2)	0.002 (2)
C18	0.057 (4)	0.071 (4)	0.049 (3)	−0.007 (3)	−0.009 (3)	0.013 (3)
C19	0.053 (4)	0.074 (4)	0.061 (4)	−0.006 (3)	−0.011 (3)	0.025 (3)
C20	0.043 (3)	0.045 (3)	0.065 (4)	−0.001 (2)	−0.006 (3)	0.016 (3)
C21	0.027 (2)	0.036 (3)	0.060 (3)	−0.0026 (19)	0.000 (2)	0.010 (2)
C22	0.022 (2)	0.028 (2)	0.064 (3)	−0.0004 (17)	0.005 (2)	0.009 (2)
C23	0.035 (3)	0.031 (3)	0.070 (4)	0.002 (2)	0.007 (2)	0.005 (2)
C24	0.046 (3)	0.029 (3)	0.069 (4)	0.000 (2)	0.010 (3)	−0.005 (2)
C25	0.042 (3)	0.039 (3)	0.053 (3)	−0.003 (2)	0.014 (2)	−0.006 (2)
C26	0.037 (3)	0.032 (2)	0.054 (3)	−0.001 (2)	0.008 (2)	0.003 (2)
C27	0.028 (2)	0.028 (2)	0.052 (3)	−0.0004 (18)	0.005 (2)	0.005 (2)
C28	0.062 (4)	0.042 (3)	0.067 (4)	0.003 (3)	0.004 (3)	−0.010 (3)
C29	0.033 (3)	0.031 (2)	0.067 (3)	−0.004 (2)	0.008 (2)	0.001 (2)
C30	0.049 (3)	0.032 (3)	0.066 (4)	0.003 (2)	0.007 (3)	0.006 (2)
C31	0.045 (3)	0.027 (2)	0.058 (3)	−0.002 (2)	0.008 (2)	0.009 (2)
C32	0.085 (5)	0.037 (3)	0.063 (4)	0.007 (3)	0.004 (3)	0.011 (3)
N1	0.033 (2)	0.0284 (19)	0.043 (2)	0.0001 (16)	0.0005 (17)	0.0037 (16)
N2	0.0235 (19)	0.037 (2)	0.050 (2)	−0.0018 (16)	0.0014 (16)	0.0063 (18)
O1	0.0344 (19)	0.043 (2)	0.048 (2)	−0.0013 (14)	−0.0061 (15)	0.0071 (15)
O2	0.0295 (17)	0.0403 (19)	0.051 (2)	0.0019 (14)	0.0007 (14)	0.0059 (15)
O3	0.0366 (18)	0.0320 (17)	0.055 (2)	−0.0009 (14)	0.0031 (15)	−0.0027 (15)
O4	0.045 (2)	0.0295 (17)	0.0461 (19)	0.0016 (14)	0.0055 (15)	0.0068 (14)
Pt1	0.02610 (9)	0.02845 (10)	0.04157 (11)	−0.00042 (6)	−0.00348 (7)	0.00636 (7)
Pt2	0.02732 (10)	0.02679 (9)	0.04527 (11)	−0.00091 (6)	0.00418 (7)	0.00340 (7)

### Geometric parameters (Å, º)

**Table:**

C9—C10	1.385 (7)	C16—H16A	0.9800
C6—C11	1.414 (7)	C16—H16B	0.9800
C10—C11	1.380 (7)	C16—H16C	0.9800
C12—C13	1.492 (8)	C17—H17	0.9500
C13—C14	1.413 (8)	C18—H18	0.9500
C14—C15	1.382 (7)	C19—H19	0.9500
C15—C16	1.508 (7)	C2—H2	0.9500
C17—C18	1.368 (8)	C20—H20	0.9500
C18—C19	1.378 (9)	C23—H23	0.9500
C1—C2	1.390 (8)	C24—H24	0.9500
C19—C20	1.381 (9)	C26—H26	0.9500
C20—C21	1.389 (7)	C28—H28A	0.9800
C21—C22	1.463 (7)	C28—H28B	0.9800
C22—C23	1.402 (7)	C28—H28C	0.9800
C23—C24	1.362 (8)	C3—H3	0.9500
Br2—C25	1.905 (5)	C30—H30	0.9500
C24—C25	1.386 (8)	C32—H32A	0.9800
C25—C26	1.388 (7)	C32—H32B	0.9800
C22—C27	1.407 (6)	C32—H32C	0.9800
C26—C27	1.388 (7)	C4—H4	0.9500
C28—C29	1.511 (7)	C7—H7	0.9500
C2—C3	1.392 (9)	C8—H8	0.9500
C29—C30	1.397 (8)	C1—N1	1.325 (6)
C30—C31	1.387 (8)	C5—N1	1.367 (6)
C31—C32	1.504 (8)	C17—N2	1.331 (7)
C3—C4	1.367 (9)	C21—N2	1.378 (6)
C4—C5	1.400 (7)	C13—O1	1.279 (6)
C5—C6	1.452 (7)	C15—O2	1.284 (5)
C6—C7	1.404 (7)	C29—O3	1.270 (6)
C7—C8	1.369 (8)	C31—O4	1.290 (6)
Br1—C9	1.915 (5)	C11—Pt1	1.970 (5)
C8—C9	1.376 (8)	N1—Pt1	1.995 (4)
C1—H1	0.9500	O1—Pt1	2.077 (3)
C10—H10	0.9500	O2—Pt1	2.007 (3)
C12—H12A	0.9800	C27—Pt2	1.969 (5)
C12—H12B	0.9800	N2—Pt2	1.999 (4)
C12—H12C	0.9800	O3—Pt2	2.081 (3)
C14—H14	0.9500	O4—Pt2	2.005 (3)
			
N1—C1—C2	122.5 (5)	C21—C20—H20	120.1
N1—C1—H1	118.8	N2—C21—C20	119.2 (5)
C2—C1—H1	118.8	N2—C21—C22	113.4 (4)
C1—C2—C3	117.8 (6)	C20—C21—C22	127.4 (5)
C1—C2—H2	121.1	C23—C22—C27	120.8 (5)
C3—C2—H2	121.1	C23—C22—C21	124.6 (4)
C4—C3—C2	119.9 (6)	C27—C22—C21	114.6 (4)
C4—C3—H3	120.0	C24—C23—C22	120.3 (5)
C2—C3—H3	120.0	C24—C23—H23	119.9
C3—C4—C5	120.2 (5)	C22—C23—H23	119.9
C3—C4—H4	119.9	C23—C24—C25	119.0 (5)
C5—C4—H4	119.9	C23—C24—H24	120.5
N1—C5—C4	119.1 (5)	C25—C24—H24	120.5
N1—C5—C6	113.4 (4)	C24—C25—C26	122.0 (5)
C4—C5—C6	127.5 (5)	C24—C25—Br2	118.9 (4)
C7—C6—C11	120.6 (5)	C26—C25—Br2	119.0 (4)
C7—C6—C5	124.2 (5)	C27—C26—C25	119.5 (5)
C11—C6—C5	115.2 (4)	C27—C26—H26	120.2
C8—C7—C6	120.5 (5)	C25—C26—H26	120.2
C8—C7—H7	119.8	C26—C27—C22	118.3 (5)
C6—C7—H7	119.8	C26—C27—Pt2	127.0 (4)
C7—C8—C9	118.2 (5)	C22—C27—Pt2	114.7 (4)
C7—C8—H8	120.9	C29—C28—H28A	109.5
C9—C8—H8	120.9	C29—C28—H28B	109.5
C8—C9—C10	123.1 (5)	H28A—C28—H28B	109.5
C8—C9—Br1	118.4 (4)	C29—C28—H28C	109.5
C10—C9—Br1	118.5 (4)	H28A—C28—H28C	109.5
C11—C10—C9	119.6 (5)	H28B—C28—H28C	109.5
C11—C10—H10	120.2	O3—C29—C30	125.6 (5)
C9—C10—H10	120.2	O3—C29—C28	115.7 (5)
C10—C11—C6	118.1 (4)	C30—C29—C28	118.7 (5)
C10—C11—Pt1	128.2 (4)	C31—C30—C29	127.4 (5)
C6—C11—Pt1	113.7 (4)	C31—C30—H30	116.3
C13—C12—H12A	109.5	C29—C30—H30	116.3
C13—C12—H12B	109.5	O4—C31—C30	127.0 (5)
H12A—C12—H12B	109.5	O4—C31—C32	113.3 (5)
C13—C12—H12C	109.5	C30—C31—C32	119.7 (5)
H12A—C12—H12C	109.5	C31—C32—H32A	109.5
H12B—C12—H12C	109.5	C31—C32—H32B	109.5
O1—C13—C14	125.3 (5)	H32A—C32—H32B	109.5
O1—C13—C12	115.3 (5)	C31—C32—H32C	109.5
C14—C13—C12	119.4 (5)	H32A—C32—H32C	109.5
C15—C14—C13	126.9 (5)	H32B—C32—H32C	109.5
C15—C14—H14	116.5	C1—N1—C5	120.5 (4)
C13—C14—H14	116.5	C1—N1—Pt1	123.8 (3)
O2—C15—C14	127.5 (5)	C5—N1—Pt1	115.7 (3)
O2—C15—C16	113.6 (5)	C17—N2—C21	120.4 (4)
C14—C15—C16	119.0 (4)	C17—N2—Pt2	124.1 (4)
C15—C16—H16A	109.5	C21—N2—Pt2	115.6 (3)
C15—C16—H16B	109.5	C13—O1—Pt1	123.8 (3)
H16A—C16—H16B	109.5	C15—O2—Pt1	124.2 (3)
C15—C16—H16C	109.5	C29—O3—Pt2	123.8 (4)
H16A—C16—H16C	109.5	C31—O4—Pt2	124.0 (3)
H16B—C16—H16C	109.5	C11—Pt1—N1	81.89 (18)
N2—C17—C18	121.9 (6)	C11—Pt1—O2	93.04 (17)
N2—C17—H17	119.1	N1—Pt1—O2	174.80 (15)
C18—C17—H17	119.1	C11—Pt1—O1	174.72 (17)
C17—C18—C19	119.3 (6)	N1—Pt1—O1	92.90 (15)
C17—C18—H18	120.3	O2—Pt1—O1	92.15 (14)
C19—C18—H18	120.3	C27—Pt2—N2	81.73 (18)
C18—C19—C20	119.6 (5)	C27—Pt2—O4	92.57 (17)
C18—C19—H19	120.2	N2—Pt2—O4	174.29 (15)
C20—C19—H19	120.2	C27—Pt2—O3	175.20 (17)
C19—C20—C21	119.7 (6)	N2—Pt2—O3	93.57 (16)
C19—C20—H20	120.1	O4—Pt2—O3	92.12 (13)
			
N1—C1—C2—C3	−0.6 (9)	C21—C22—C23—C24	177.1 (5)
C1—C2—C3—C4	−0.2 (9)	C22—C23—C24—C25	0.6 (8)
C2—C3—C4—C5	0.4 (9)	C23—C24—C25—C26	1.9 (8)
C3—C4—C5—N1	0.1 (7)	C23—C24—C25—Br2	−175.1 (4)
C3—C4—C5—C6	178.0 (5)	C24—C25—C26—C27	−2.4 (8)
N1—C5—C6—C7	178.2 (4)	Br2—C25—C26—C27	174.6 (4)
C4—C5—C6—C7	0.2 (8)	C25—C26—C27—C22	0.3 (7)
N1—C5—C6—C11	−2.2 (6)	C25—C26—C27—Pt2	−178.7 (4)
C4—C5—C6—C11	179.8 (4)	C23—C22—C27—C26	2.1 (7)
C11—C6—C7—C8	−0.4 (7)	C21—C22—C27—C26	−177.6 (4)
C5—C6—C7—C8	179.2 (5)	C23—C22—C27—Pt2	−178.8 (3)
C6—C7—C8—C9	−0.6 (8)	C21—C22—C27—Pt2	1.5 (5)
C7—C8—C9—C10	1.2 (8)	O3—C29—C30—C31	−0.6 (9)
C7—C8—C9—Br1	−179.5 (4)	C28—C29—C30—C31	179.8 (5)
C8—C9—C10—C11	−0.9 (8)	C29—C30—C31—O4	0.5 (9)
Br1—C9—C10—C11	179.8 (3)	C29—C30—C31—C32	178.3 (5)
C9—C10—C11—C6	−0.1 (7)	C2—C1—N1—C5	1.2 (8)
C9—C10—C11—Pt1	179.7 (4)	C2—C1—N1—Pt1	179.9 (4)
C7—C6—C11—C10	0.7 (7)	C4—C5—N1—C1	−0.9 (7)
C5—C6—C11—C10	−178.9 (4)	C6—C5—N1—C1	−179.1 (4)
C7—C6—C11—Pt1	−179.1 (4)	C4—C5—N1—Pt1	−179.7 (3)
C5—C6—C11—Pt1	1.3 (5)	C6—C5—N1—Pt1	2.1 (5)
O1—C13—C14—C15	−3.6 (9)	C18—C17—N2—C21	−0.8 (8)
C12—C13—C14—C15	175.8 (5)	C18—C17—N2—Pt2	179.5 (4)
C13—C14—C15—O2	3.9 (9)	C20—C21—N2—C17	0.9 (7)
C13—C14—C15—C16	−174.6 (5)	C22—C21—N2—C17	179.7 (4)
N2—C17—C18—C19	0.4 (9)	C20—C21—N2—Pt2	−179.4 (4)
C17—C18—C19—C20	−0.1 (9)	C22—C21—N2—Pt2	−0.6 (5)
C18—C19—C20—C21	0.3 (8)	C14—C13—O1—Pt1	0.3 (7)
C19—C20—C21—N2	−0.6 (8)	C12—C13—O1—Pt1	−179.0 (4)
C19—C20—C21—C22	−179.2 (5)	C14—C15—O2—Pt1	−0.7 (7)
N2—C21—C22—C23	179.7 (4)	C16—C15—O2—Pt1	177.9 (3)
C20—C21—C22—C23	−1.7 (8)	C30—C29—O3—Pt2	−1.2 (7)
N2—C21—C22—C27	−0.6 (6)	C28—C29—O3—Pt2	178.4 (3)
C20—C21—C22—C27	178.0 (5)	C30—C31—O4—Pt2	1.5 (7)
C27—C22—C23—C24	−2.6 (7)	C32—C31—O4—Pt2	−176.5 (4)

### Hydrogen-bond geometry (Å, º)

**Table:**

*D*—H···*A*	*D*—H	H···*A*	*D*···*A*	*D*—H···*A*
C1—H1···O1	0.95	2.40	2.999 (7)	121
C4—H4···O4^i^	0.95	2.58	3.281 (6)	131
C17—H17···O3	0.95	2.45	3.034 (6)	120
C17—H17···Br1^ii^	0.95	2.87	3.693 (6)	145

Symmetry codes: (i) −*x*+1/2, −*y*+1/2, −*z*+1; (ii) *x*−1/2, −*y*+1/2, *z*+1/2.

## References

[bb1] Bondi, A. (1964). *J. Phys. Chem.* **68**, 441–451.

[bb2] Bruker (2014). *APEX2*, *SADABS* and *SAINT*. Bruker AXS Inc., Madison, Wisconsin, USA.

[bb3] Chi, Y. & Chou, P.-T. (2010). *Chem. Soc. Rev.* **39**, 638–655. 10.1039/b916237b 20111785

[bb4] Cockburn, B. N., Howe, V., Keating, T., Johnson, B. F. G. & Lewis, J. (1973). *J. Chem. Soc. Dalton Trans.* pp. 404–410.

[bb5] Farrugia, L. J. (2012). *J. Appl. Cryst.* **45**, 849–854.

[bb6] Hudson, Z. M., Blight, B. A. & Wang, S. (2012). *Org. Lett.* **14**, 1700–1703. 10.1021/ol300242f 22414237

[bb7] Liu, J., Yang, C.-J., Cao, Q.-Y., Xu, M., Wang, J., Peng, H.-N., Tan, W.-F., Lü, X.-X. & Gao, X.-C. (2009). *Inorg. Chim. Acta*, **362**, 575–579.

[bb8] Ma, D.-L., He, H.-Z., Leung, K.-H., Chan, D. S.-H. & Leung, C.-H. (2013). *Angew. Chem. Int. Ed.* **52**, 7666–7682. 10.1002/anie.201208414 23765907

[bb9] Macrae, C. F., Bruno, I. J., Chisholm, J. A., Edgington, P. R., McCabe, P., Pidcock, E., Rodriguez-Monge, L., Taylor, R., van de Streek, J. & Wood, P. A. (2008). *J. Appl. Cryst.* **41**, 466–470.

[bb10] Sheldrick, G. M. (2008). *Acta Cryst.* A**64**, 112–122. 10.1107/S0108767307043930 18156677

[bb11] Sheldrick, G. M. (2015). *Acta Cryst.* C**71**, 3–8.

